# Multi–Joint Angles Estimation of Forearm Motion Using a Regression Model

**DOI:** 10.3389/fnbot.2021.685961

**Published:** 2021-08-02

**Authors:** Zixuan Qin, Sorawit Stapornchaisit, Zixun He, Natsue Yoshimura, Yasuharu Koike

**Affiliations:** ^1^Department of Information and Communications Engineering, Tokyo Institute of Technology, Yokohama, Japan; ^2^Institute of Innovative Research, Tokyo Institute of Technology, Yokohama, Japan; ^3^Precursory Research for Embryonic Science and Technology (PRESTO), Japan Science and Technology Agency (JST), Saitama, Japan

**Keywords:** convolutional neural networks, geometry plot, regression model, surface electromyography, transfer learning

## Abstract

To improve the life quality of forearm amputees, prosthetic hands with high accuracy, and robustness are necessary. The application of surface electromyography (sEMG) signals to control a prosthetic hand is challenging. In this study, we proposed a time-domain CNN model for the regression prediction of joint angles in three degrees of freedom (3-DOFs, include two wrist joint motion and one finger joint motion), and five-fold cross validation was used to evaluate the correlation coefficient (CC). The CC value results of wrist flexion/extension motion obtained from 10 participants was 0.87–0.92, pronation/supination motion was 0.72–0.95, and hand grip/open motion was 0.75–0.94. We backtracked the fully connected layer weights to create a geometry plot for analyzing the motion pattern to investigate the learning of the proposed model. In order to discuss the daily updateability of the model by transfer learning, we performed a second experiment on five of the participants in another day and conducted transfer learning based on smaller amount of dataset. The CC results improved (wrist flexion/extension was 0.90–0.97, pronation/supination was 0.84–0.96, hand grip/open was 0.85–0.92), suggesting the effectiveness of the transfer learning by incorporating the small amounts of sEMG data acquired in different days. We compared our CNN-based model with four conventional regression models, the result illustrates that proposed model significantly outperforms the four conventional models with and without transfer learning. The offline result suggests the reliability of the proposed model in real-time control in different days, it can be applied for real-time prosthetic control in the future.

## Introduction

The human hand plays a crucial role in many activities of daily living (ADL), therefore, loss of the upper extremity could significantly impact functional independence (National Academies of Sciences, 2017). To support the ADL of amputees, prosthetic hands were developed, which can mimic human-hand motions to complete activities. Several studies have investigated the possibility of controlling the prosthetic hand using surface electromyography (sEMG) signals because the use of sEMG signals can directly utilize human neural pathways to restore ADL function (Scheme and Englehart, 2011). For abled people, hand movements occur due to the contraction and relaxation of the forearm muscles controlled by the brain. When the forearm is lost, sEMG signals of the remaining muscle or electroencephalography (EEG) signal can be considered as a control signal for prosthetic hands. When compared to EEG signals, sEMG signals have higher accuracy and reliability (Scheme and Englehart, 2011; Farina et al., 2014; Xia et al., 2018). However, the method of using bio-signals (such as EEG or sEMG signals) to control a robotic hand is still a challenge.

Many studies considered using pattern recognition via conventional machine learning methods, such as support vector machine (SVM), to perform motion classification (Yoshikawa et al., 2006; Angkoon et al., 2012). Some researches trained regression predictors using deep learning methods, such as recurrent neural networks (RNNs), convolutional neural networks (CNN), even a combination of RNNs and CNNs (recurrent convolutional neural networks, RCNN) (Atzori et al., 2016; Wang et al., 2016; Hu et al., 2018; Xia et al., 2018).

Some researchers have used CNN-based models to perform activity recognition and motion classification. Jiang and Yin (2015) proposed a deep convolutional neural network (DCNN) to learn the optimal features from activity images for motion recognition, and obtained high recognition accuracy. Rehman et al. (2018) found that CNN can be used to recognize sEMG patterns for long-term classification even though the sEMG signal is not stable. They extracted four time-domain features and compared them with classical machine learning methods, such as linear discriminant analysis (LDA), stacked sparse autoencoders with features (SSAE-f), and SSAE with raw samples (SSAE-r). CNN was found to be better. Huang and Chen (2019) proposed a hybrid structure combining CNN with LSTM to form a CNN–LSTM model and used the time-frequency domain feature extracted from the EMG signal to classify hand movements. The model was compared with the physical features of conventional methods such as SVM with spectrogram. Ameri et al. (2019) developed a regression-based CNN network for online EMG estimation of wrist motions and compared them with SVM. Bai et al. (2021) proposed a hybrid CNN-LSTM model that effectively combines feature extraction and time series regression for deep learning using sEMG to recognize hand gesture, the experiment results shown that the EMG signal processed by Fast Fourier Transform (FFT) as the characteristic value has better performance, and complex gesture signals can be accurately predicted. In addition to classifiers, CNN is also widely used in regression analysis of hand motions. Koch et al. (2020) chose RNNs to do hand movements regression from sEMG signal, the results proved that even with the relatively simple networks the hand gestures can be regressed quite accurately. Bao et al. (2021a) proposed a CNN-LSTM framework named Deep Kalman Filter Network (DKFN) to estimate wrist and finger kinematics using sEMG, the results shown 0.6–0.8 R^2^ result of fingers kinematics and 0.7–0.9 R^2^ of wrist kinematics. However, all the studies that used CNN to do classification or regression analysis without discussing the learning methodology of the model from sEMG signals. Moreover, in sEMG pattern recognition, the model accuracy decreases in another day due to the electrode shift or skin impedance, therefore, updating the control system for different day is significant.

Nowadays, modern researches start to use transfer learning to deal with the possible changes in sEMG signals in different days, because transfer learning requires only a short training session to recalibrate the system for new sEMG signals. The new source of information can be leveraged to build a more precise and reliable classifier or regression model for a new dataset, transfer learning allows the capture of more general and robust features, so that the model is then able to use these general features to build a more effective performance of a new sEMG activity. Côté-Allard et al. (2017) combined CNN with transfer learning to classify hand gestures from sEMG, and the model was robust and achieved average classification accuracy of 97.81% on seven gestures. Ameri et al. (2020) proposed an approach based on CNNs with transfer learning to overcome the lack of robustness to confounding factors in EMG pattern recognition-based control, including CNN classification and regression model. Thus, transfer learning not only has effect on classification model, but also can be applied to regression prediction. (Bao et al., 2021b) proposed a state-of-the-art transfer learning method with regression supervised domain adaptation (SDA) for wrist kinematics estimation using sEMG signal, effectively reduced the burden of regular model re-training/recalibration under domain shift effects. In our work, we considered to use transfer learning for checking the proposed model robustness in different days.

Although it is difficult to discuss the weight of the CNN layers for analyzing muscle anatomy and motion activation, Stapornchaisit et al. (2019) used a topology graph to plot the weight of the independence component (IC) to investigate the relationship between the weight of each channel. Thus, we can attempt an analysis of the movement area on both sides of the human forearm by backtracking from the weight parameters in the final layer of the proposed model.

In this research, we proposed a high-accuracy CNN-based regression model to predict forearm joint angles include two wrist motions and one finger motion, and discussed the learnability and high stability of the proposed model for amputees to update the parameters every day. In our experiment, sEMG data and joint angles were collected from participants at the same time as data. As for the sEMG signal, the muscle activities which related to wrist motion and finger motion are mixed, it is difficult to separate these motion. Thus, we designed three degrees-of-freedom (3-DOFs, include wrist flexion/extension and pronation/supination as wrist motions and hand grip/open as finger motion) joint angles as the output of the proposed CNN model based on multi-array sEMG input, the output of the three joint angles will be sent to prosthetic hand for controlling in the future. Then, we used the backtracking method to create a geometry plot for muscle area analysis. We designed a second experiment for smaller amount of dataset and checked that whether transfer learning can improve the regression prediction accuracy. We compared our model with four conventional regression model with and without transfer learning respectively, the conventional regression models are: linear regression (LR), support vector regression (SVR), k-nearest neighbors (KNN) and decision tree regression (DT), and the model comparison results proved that proposed model significantly outperforms conventional regression models. This paper shows the stability and superiority of the proposed model, and we consider it can be applied for future real-time prosthetic hand control.

## Materials and Methods

### Participants

Ten right-handed participants (S1–S10) with intact limbs participated in this study; they were nine males and one female, aged 21–43. Data from the participants were acquired at the Tokyo Institute of Technology, Japan. The information of the participants is presented in Table 1. The study protocol was approved by the ethics committee of the Tokyo Institute of Technology and was conducted in accordance with the Declaration of Helsinki. All participants were asked to read the participant information sheet and provide written informed consent to participate in the study.

**Table 1:** Participant information.

**Participant ID**	**Age(years)**	**Handedness**	**Gender**
S1	24	Right	Male
S2	23	Right	Male
S3	21	Right	Male
S4	23	Right	Female
S5	23	Right	Male
S6	26	Right	Male
S7	43	Right	Male
S8	25	Right	Male
S9	24	Right	Male
S10	25	Right	Male

### sEMG Data Acquisition

**Figure 1:**
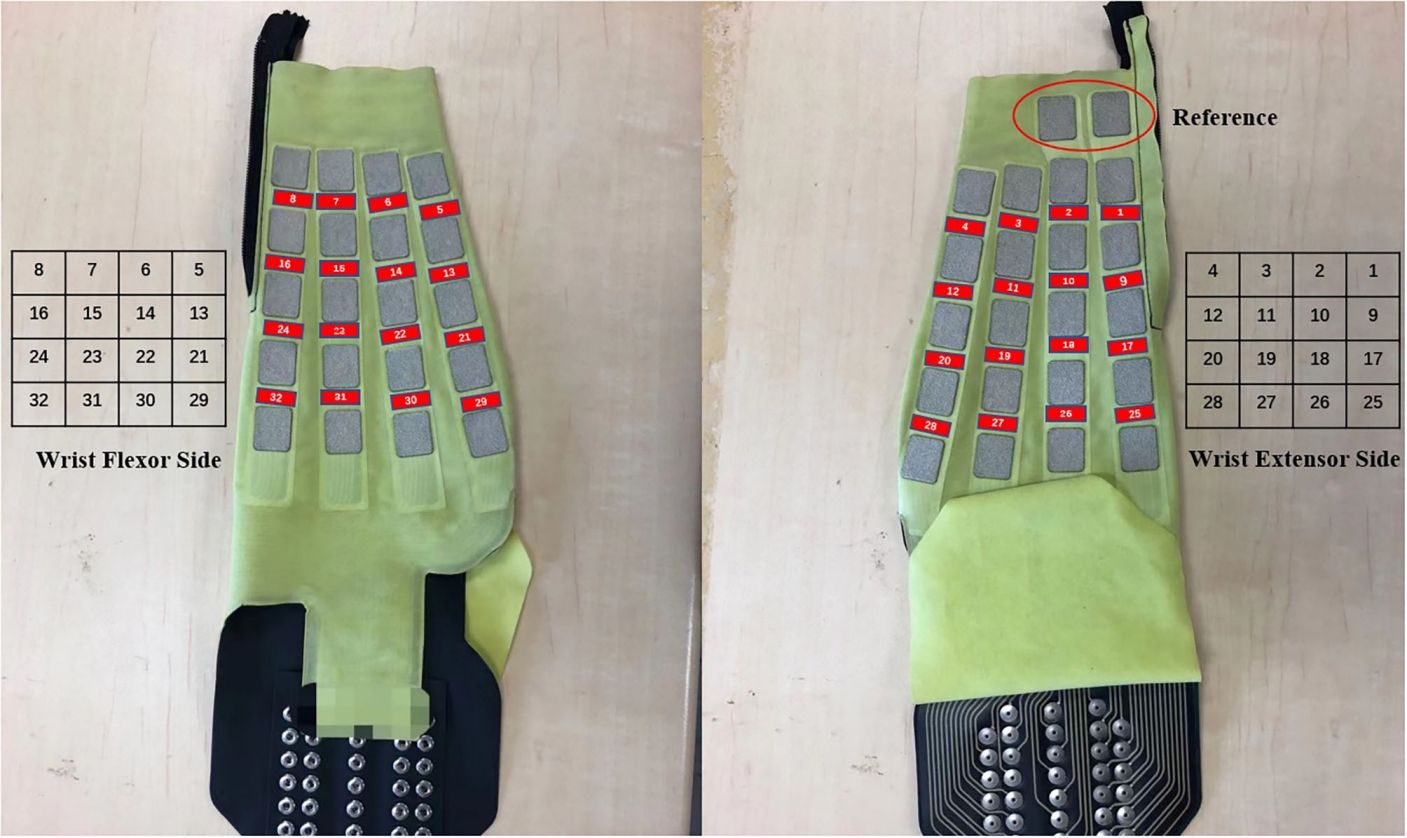
Multi-array electrode forearm sleeve (32 channels, right-handed). The red parts show the positions of 32 channels. Left: Wrist flexor side after wearing the sleeve, includes ch5–ch8, ch13–ch16, ch21–ch24, and ch29–ch32. Right: Wrist extensor side after wearing the sleeve, includes ch1–ch4, ch9–ch12, ch17–ch20, and ch25–ch28. The matrix represents the channel distribution on multi-array electrode sleeve and the number represents the channel number. Geometry plot for motion analysis is based on the two matrices.

We used a bipolar multi-array electrode with 32 channels (Yasuharu et al., 2020) to acquire EMG signals (SMK Corp., SEIREN Co., Ltd.). Figure 1 shows the multi-array electrode sleeve, the left and right figures show the wrist flexor and wrist extensor sides, respectively. There are 40 electrodes (5 × 8), each five electrodes measure four channels (two electrodes are shared by one channel), and one reference. The flexor side consists of 16 channels, including ch5–ch8, ch13–ch16, ch21–ch24, and ch29–ch32. The extensor side consists of 16 channels, including ch1–ch4, ch9–ch12, ch17–ch20, and ch25–ch28. The two sides are arranged as a 4 × 4 matrix, and the channel numbers help us to understand the position of each channel. The ADC resolution of the multi-array electrode is 16 bit. The sEMG signal was sent to PC by a Bluetooth module. We acquired raw sEMG signal from this system that consists of a dataset in our experiment, and lab streaming layer (LSL) was used to synchronize data. Before the experiment, we use water to make the sensors and participants' skin perfectly fit, and check the sEMG signal quality. We chose 500 Hz as the sampling frequency (Yasuharu et al., 2020) due to the limited signal transmission speed of Bluetooth low energy, and we do not need to adjust the electrode location for each participant.

### Joint Angle Acquisition

**Figure 2:**
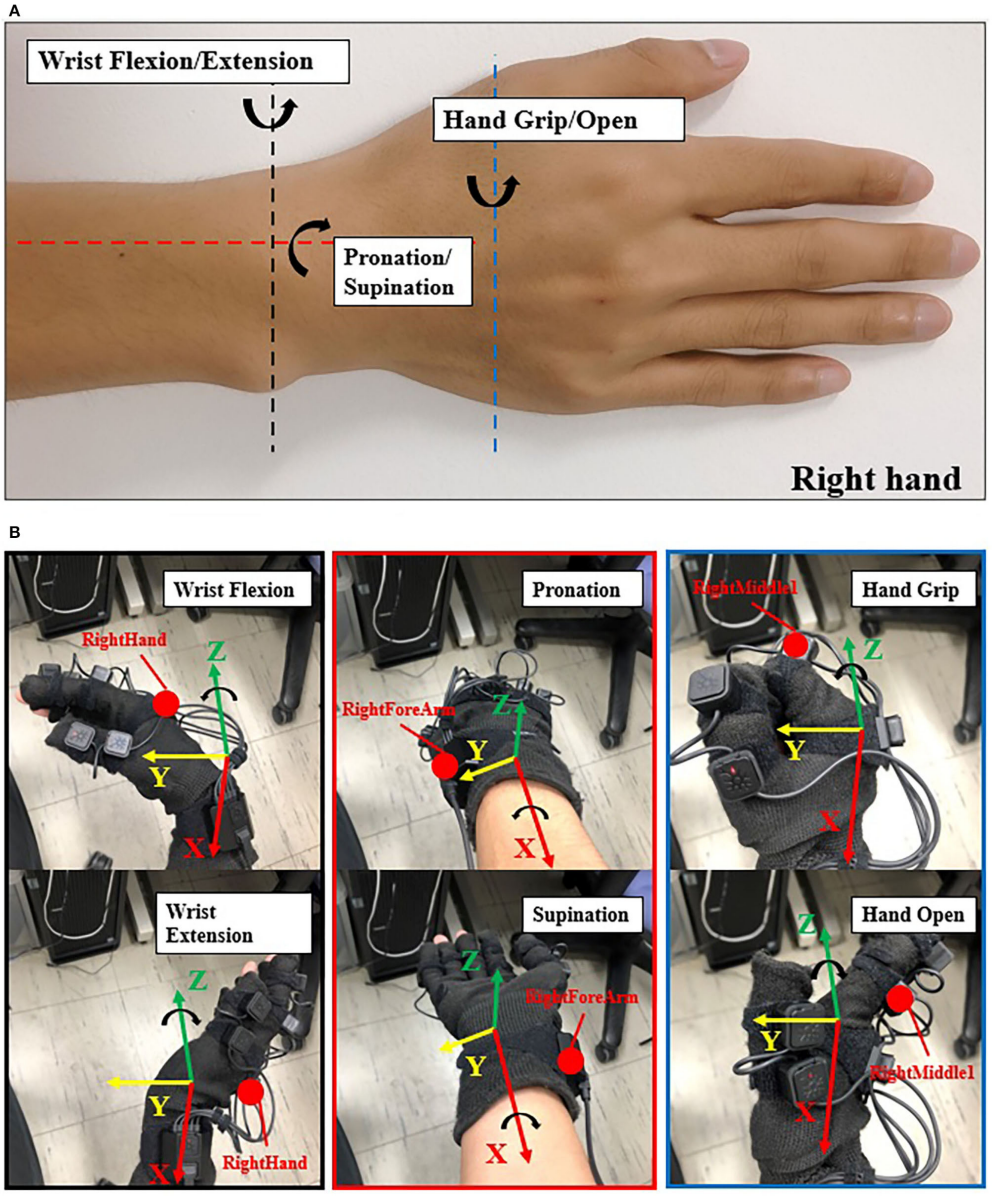
Illustration of joint angle data collection. **(A)** The 3-DOFs movements (right hand) in this study: black dash line shows the DOF of wrist flexion (WF) and wrist extension (WE); red dash line shows the DOF of pronation (P) and supination (S); blue dash line shows the DOF of hand grip (HG) and hand open (HO). WF/WE and P/S are wrist motion, HG/HO is finger motion. **(B)** Data collection with Perception Neuron Motion Capture Glove with 9 markers (right hand). The red point here shows the focus markers we chose. While performing the experiment, we chose “RightForeArm” marker to acquire joint angles of pronation and supination (P/S), “RightHand” marker to acquire joint angles of wrist flexion and wrist extension (WF/WE), and “RightHandMiddle1” to acquire joint angles of hand grip and hand open (HG/HO) motion. The coordinate here shows the BVH global coordinates, red axis is X axis, yellow axis is Y axis, and green axis is Z axis. WF/WE angles are obtained based on “RightHand” marker rotating around the Z axis; P/S angles are obtained based on “RightForeArm” marker rotating around the X axis; and HG/HO angels are obtained based on “RightMiddle1” marker rotating around the Z axis. Counterclockwise rotation shows positive angle and clockwise rotation shows negative angle, i.e., WF, P, and HG angles are positive values, and WE, S and HO angles are negative values.

We used the Perception Neuron Motion Capture system (Noitom Ltd., China) to collect joint angles when participants performed specified motions. This system is a type of motion capture device that is applied to movement analysis for film makers, game developers, sports analysis, and biomechanics research related to this work (Kim et al., 2019). In our work, we used only the right hand and right forearm parts. Each participant was required to wear the glove with motion capture markers (Figure 2B) and complete calibration to build a skeleton model on the Axis Neuron software provided by Noitom Corp. After successful calibration, we can check the rotation angles by choosing the specific name such as “RightHand,” etc. (Yuanhui, 2014) and axis of rotation (X, Y, or Z axis).

While using the Axis Neuron, we chose the BVH data type as the data format, which includes joint hierarchy and movement data. The sensory data of each joint were stored in the corresponding joint local coordination system, body joints of Perception Neuron were arranged as tree structures, and children joints were connected corresponding to their parent joint (Chen et al., 2017). Each joint angle data in the BVH data is calculated from the children joint coordination system to the parent joint coordinate system by internally multiplying the transformational matrix. Finally, the data are shown in global coordinates.

Figure 2A shows the 3-DOFs movements in our study (right hand): black dash line shows the DOF of wrist flexion (WF) and wrist extension (WE); red dash line shows the DOF of pronation (P) and supination (S); blue dash line shows the DOF of hand grip (HG) and hand open (HO). WF/WE and P/S are wrist motion, HG/HO is finger motion. Figure 2B shows the required markers (the red points,: RightForeArm, RightHand, and RightMiddle1) to acquire the joint angles, “RightHand” marker was to acquire the joint angles of WF and WE motion, “RightForeArm” marker was to acquire the joint angles of P and S motion, and “RightHandMiddle1” marker was to acquire the joint angles of HG and HO motion.

According to the Neuron Coordinate document provided by Noitom Ltd. and Aiuto Co. Ltd., Japan (Aiuto Co., Ltd., 2020), the global coordinates of the BVH data are shown in Figure 2B, where the green, red, and yellow axes are the Z-axis, X-axis, and Y-axis, respectively. Thus, WF/WE joint angles are obtained based on “RightHand” marker rotating around the Z axis, P/S joint angles are obtained based on “RightForeArm” marker rotating around the X axis, and HG/HO joint angles are obtained based on “RightMiddle1” marker rotating around the Z axis. The value of the angle is positive when the rotation is counterclockwise and negative when the rotation is clockwise. Therefore, the joint angles of WF, P, and HG are positive angles, and that of WE, S, and HO are negative angles.

**Figure 3:**
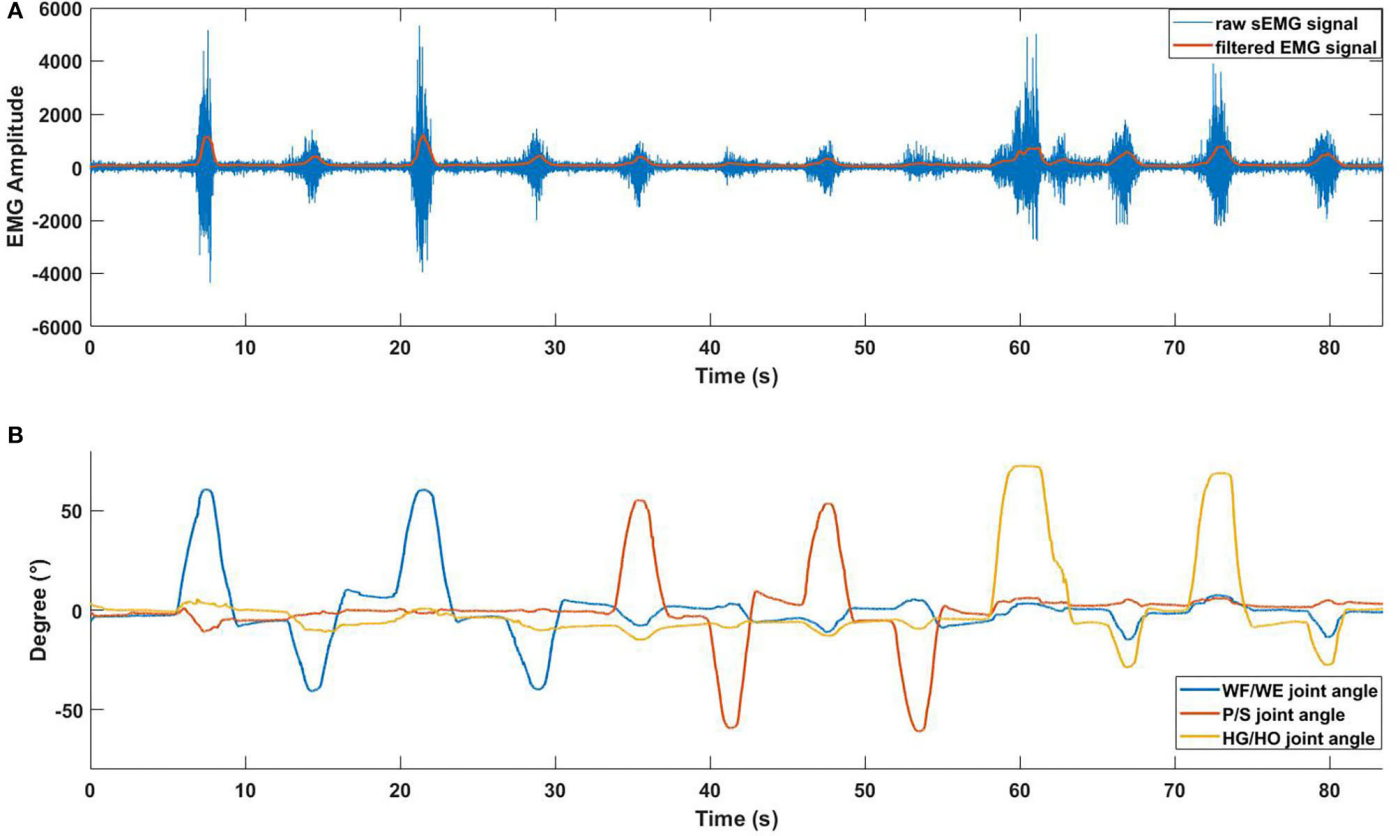
EMG signal with joint angle data (participant S1). **(A)** Pre-processed sEMG signal with raw sEMG signal. The data is obtained from ch10. Blue line represents raw sEMG signal, orange line represents filtered EMG signal before the normalization. The horizontal axis represents the duration time of one trial, vertical axis represents the amplitude of sEMG signal. **(B)** Collected 3-DOF joint angle data using Perception Neuron Motion Capture system.

The sampling rate of data collection from the Perception Neuron Motion Capture system was 120 Hz. During the experiment, 3-DOF joint angles and sEMG signal were collected at the same time and synchronized by LSL system. Figure 3 shows an example of the synchronization between sEMG signal and joint angles.

### Experiment Protocol and Data Processing

**Figure 4:**
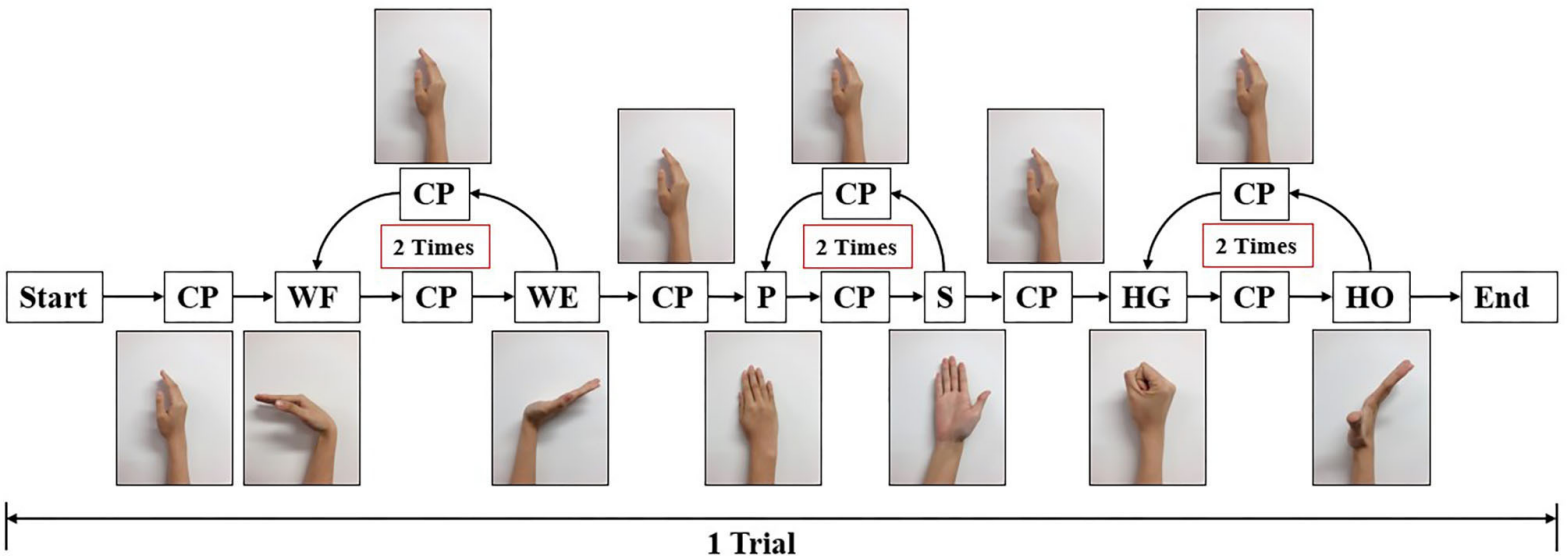
Experiment paradigm for one trial. In each trial, participant should start from central position (CP), perform wrist flexion (WF) and wrist extension (WE) two times, pronation (P) and supination (S) two times, and hand grip (HG) and hand open (HO) two times. After each motion, they should move their forearm to CP and then perform the next motion. The screen displays the next motion to be performed.

Participants (Table 1) were asked to sit on a chair in front of a screen. After calibrating the joint angles on Perception Neuron Motion Capture system, we checked both the quality of joint angles and sEMG signal. Before the experiment, each participant underwent trial sessions to familiarize themselves with the experimental process (Figure 4). After completing each trial, the participants were asked to relax for approximately 2 min before the next trial to prevent fatigue.

For ensuring long-term stability that can be used in the real-time control of a prosthetic hand, we must ensure high accuracy and robustness in another day. We invited the 10 participants to conduct experiment, and five of them were invited to do the experiment again with smaller amount of dataset. Transfer learning was applied to solve this problem, this process is called *the second experiment* in this paper. In order to distinguish the second experiment from the previous experiment (with 10 participants and larger dataset), we call the previous experiment *initial experiment*.

During the initial experiment, we acquired sEMG signal and joint angles at the same time. Each participant performed 10 trials, the following order of each trail shown as follows: WF/WE twice, P/S twice, and HG/HO twice. At the beginning of the experiment, participants performed their hand as a central position (CP). The screen then displayed the movement to be performed, and the participants had to rotate their forearm or finger joint from the CP as per the displayed movements. For each motion, participants rotated their hand to the maximum angle that can be reached, and rotated it back to the CP. The next motion is performed as per the following animation. For example, for WF/WE, the order of motion should be as follows: CP-WF-CP-WE-CP. Figure 2B shows a participant performing the experiment and motions according to the animation on the screen. The experimental paradigm was created using MATLAB (The MathWorks, Inc., USA). The second experiment also used the same experimental paradigm, totally 5 trials to reduce the dataset amount.

After the data acquisition step, we used the filter proposed in (Koike and Kawato, 1995) to process the absolute value of the raw sEMG signal to the integrated EMG (IEMG) signal, and normalized the IEMG signal from 0 to 1. Figure 3 shows the pre-processed result of the raw sEMG signal and the synchronized joint angle data (from participant S1). In Figure 3A, blue line is the raw sEMG (ch10), and orange line is the filtered EMG signal. Combining Figures 3A,B, we can see clear data synchronization between EMG and angle data *via* LSL time stamps. Owing to the difference in sampling rate between the filtered sEMG signal and joint angle data, we resampled the sEMG signal data from 500 Hz to 120 Hz to match angle data from perception neuron.

To aggregate as dataset, we used a window of 500 ms to segment sEMG data and joint angles, and the ideal dataset should be a 60 × 32 matrix corresponding to a 1 × 3 vector with 3-DOFs joint angles. Hence, we calculated the mean value of joint angles as target angles in the dataset. The interval of the sliding window is 100 ms, which means a 400 ms overlap of the data. After segmenting all the 10 trials of EMG data and joint angle data, for the initial experiment, we randomly combined the 10 trials data into five groups of datasets to perform five-fold cross validation. Then, the 10 trials data were aggregated into five groups of time-domain datasets. For the second experiment, there are 5 trails, one trial data is considered as a group with a total of five groups for the five-fold CV.

### CNN-Based Regression Model

**Figure 5:**
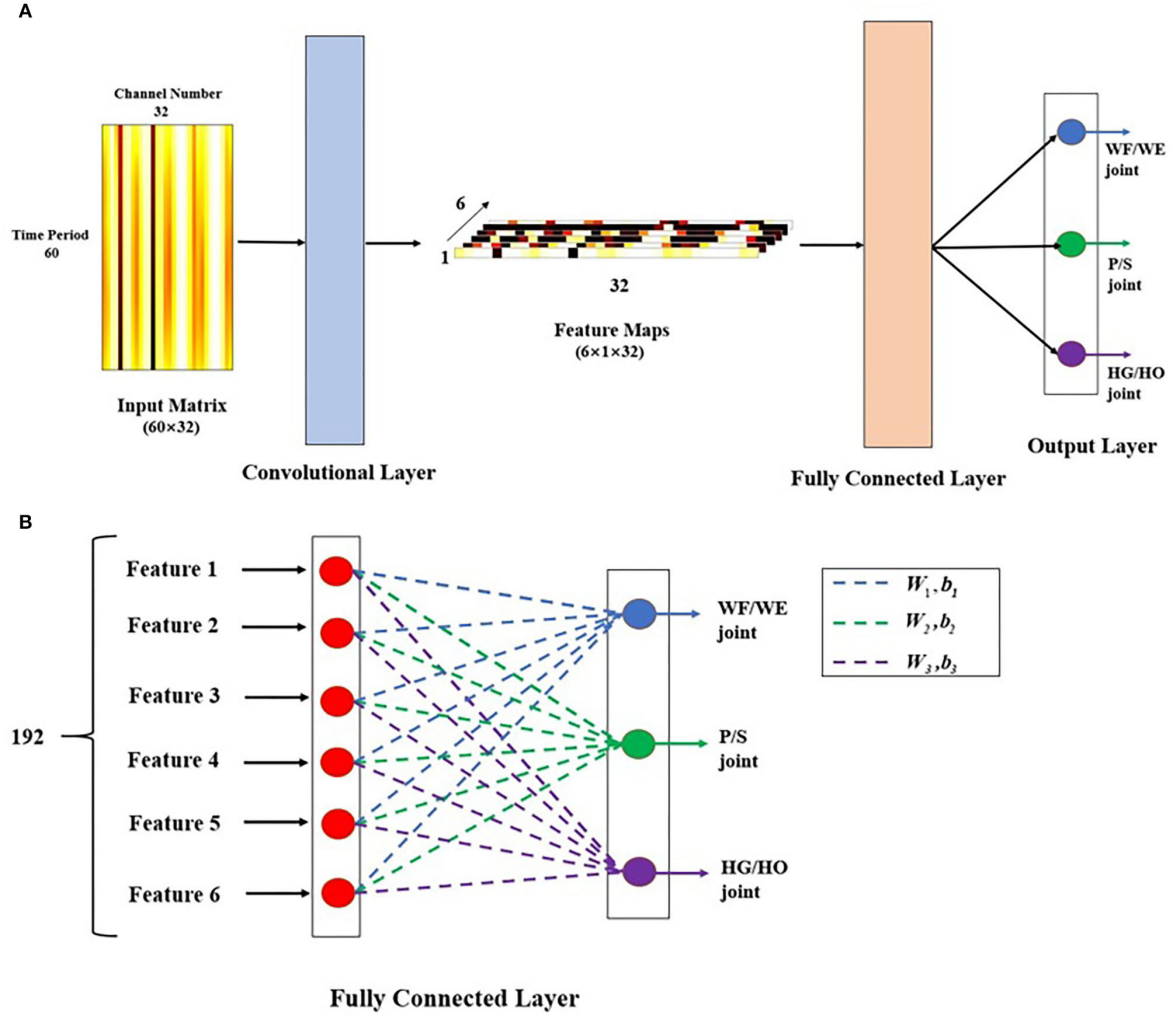
Proposed CNN-based regression model. **(A)** Model structure. This model includes two layers: convolutional layer and fully connected (FC) layer. The input matrix is 500 ms time-domain EMG signal in 32 channels, as a 60 × 32 image. Here, the image is the filtered EMG signal from a participant performing the WF motion. In the convolutional layer, there are six 60 × 1 filters, stride = 1, padding = 0, and activation function is tanh. The output after convolutional layer is six 1 × 32 feature maps, then go through FC layer directly to get three joint angles as 3 × 1 vector as output of this model. **(B)** Details description of FC layer. After the six feature maps were sent to FC layer. They connect end to end to become a 192 × 1 sequence. Each red point shows one transposed feature map with 32 × 1 represents a neuron. The sequence performs dot multiplication on three different weight (***W***) and sum the corresponding bias (***b***) to obtain different joint angles. Blue dashed line represents ***W***_**1**_ and ***b***_**1**_, which is related to WF/WE joint angle, green dashed line represents ***W***_**2**_ and ***b***_**2**_, which is related to P/S joint angle, and purple dashed line represents ***W***_**3**_ and ***b***_**3**_, which is related to HG/HO joint angle.

CNN is the main architecture of deep learning for a multi-array of data, such as images, signals, and languages. CNN works on the local receptive field, shared weights, and pooling (Le, 2015). In this work, we proposed a regression model for joint angle estimation based on CNN (Figure 5A). This model includes two layers: a convolutional layer and a fully connected (FC) layer. Similar to the conventional two-dimensional (2D) CNN, the input is a time-domain matrix data, which we regard as a 2D image. The input size is 60 × 32, where 60 represents the number of input sample that is 500 ms (the sampling rate of the processed EMG signals and joint angles are 120 Hz); 32 represents the number of channels.

In the convolutional layer, we designed the architecture as a channel-wise CNN (CW-CNN), which was mentioned in (Sakhavi et al., 2018), Discussion section will discuss the choice of the convolutional layer. There are six channel-wise filters, and each filter size is 60 × 1 in order to compress the time period dimension from 60 to 1. The outputs of the convolutional layer are six 1 × 32 vector feature maps such that we can use backtrack to easily analyze each channel. The activation function in this layer is a tanh function, without any padding and max pooling layer, and with a stride size of 1.

After the convolutional layer, the feature maps directly go to the FC layer (Figure 5B). There are six 1 × 32 feature maps, which are transposed and connected end-to-end to become a 192 × 1 vector. There are three groups of weight (W) and bias (b) in this layer, each of which can be used to calculate one joint angle. The 192 × 1 vector input was used to obtain different joint angle outputs using (1).

(1) $$\begin{array}{l} {Angle}_{i}=W_{i}^{T}\cdot fm+b_{i} \end{array}$$

where *i* is the joint number and *i* = 1,2,3; **Angle**_**i**_ is the joint angle; **Angle**_**1**_ is the WF/WE joint angle; **Angle**_**2**_ is the P/S joint angle; **Angle**_**3**_ is the HG/HO joint angle; **fm** is the feature map, which is a fully connected feature map with a size of 192 × 1; and **W**_**i**_ is the FC layer weight. Among them, **W**_**1**_ is related to the WF/WE joint, **W**_**2**_ is related to the P/S joint, and **W**_**3**_ is related to the HG/HO joint. ${\text{W}}_{\text{i}}=\left\{{\text{w}}_{\text{i}}^{\text{j}}\right\}$, where *j* = 1,2,…,6 corresponds to one feature map **fm**^**j**^ with 32 weight numbers, and **b**_**i**_ is a bias. After the FC layer, the output of the regression model is a 3 × 1 vector, which includes three joint angles: WF/WE, P/S, and HG/HO. The reason to choose the 3-DOFs angles as model output is that, in the future control of prosthetic hand, the three joint angles will be regarded as control command and sent to the three motors respectively in prosthetic hand directly, so that to rotate each motor to the corresponding angular position.

For the proposed CNN-based model, each trial was individually trained to use k-fold cross-validation (k-fold CV) (Stone, 1974; Rodríguez et al., 2010). Cross validation is used to evaluate the predictive performance of a model, particularly the performance of the trained model on new data, which can reduce overfitting to a certain extent. Further, more effective information can be obtained from limited data. The k-fold CV reduces the variance by averaging the results of k different group trainings; hence, the performance of the model is less sensitive to the division of data. In this study, *k* = 5, *i.e*., we trained and tested the dataset using a five-fold CV.

To evaluate the model using five-fold CV, we use the correlation coefficient (CC), which is used to statistically measure the strength of the relationship between two variables (Taylor, 1990). For example, in machine learning, CC is used to measure the relationship strength between the estimated angle series and measured angle series in a dataset. CC values range between −1.0 and 1.0, a correlation of 1.0 shows a perfect positive correlation and −1.0 shows a negative correlation; CC = 0.0 shows no linear relationship between the two series. Therefore, if we want to train an ideal rotation angle predictor model, we should obtain the CC result as approximately 1.0, i.e., for the predictions and measurements to be positively correlated at a high level. The equation of CC is shown in (2).

(2) $$CC=\frac{\sum_{i=1}^{n}\left(X_{i}-\bar{X}\right)(Y_{i}-\bar{Y})}{\sqrt{\sum_{i=1}^{n}{\left(X_{i}-\bar{X}\right)}^{2}}\sqrt{\sum_{i=1}^{n}{\left(Y_{i}-\bar{Y}\right)}^{2}}}$$

where *X* and *Y* are two series variables and *n* is the number of samples. In this study, the dataset was divided into five-folds. In every iteration, the proposed regression model was learned using four-folds. The remaining fold was tested to calculate performance indicators such as CC. Thus, we obtained five evaluation values after the five-fold CV of a motion pattern, and the average of the five results of each fold was calculated to obtain the final testing result (Diamantidis et al., 2000; Sakhavi et al., 2018), as shown in (3).

(3) $$CC_{5}=\frac{1}{5}\sum_{k=1}^{5}CC_{k}$$

### Geometry Plot of FC Layer Weight

**Figure 6:**
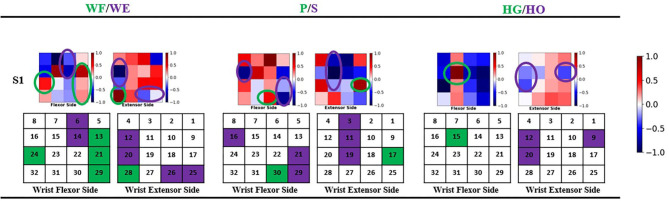
Geometry plot of participant S1. Each group of geometry plot shows flexor side (left) and extensor side (right). The color bar shows the weight normalized from −1 to 1. White color shows the channel does not contribute to motions, red color shows 0–1 and contributes to positive joint angles (WF, P, HG), and blue color shows −1–0 and contributes to negative joint angles (WE, S, HO). The circles show the motion pattern position: green circle shows the motion pattern corresponding to positive joint angles, and purple circle shows the motion pattern corresponding to negative joint angels. Under the geometry plot, the matrix shows the motion pattern color with channel index.

The geometry plot shows the weight of the feature map (Stapornchaisit et al., 2019), which has a significant contribution to the different forearm motions in the FC layer. Here, the weights of the FC layer were separated into two parts and distributed corresponding to channels (Figure 1) representing the wrist flexor side and wrist extensor side, respectively. As previously mentioned, in the FC layer, the size of weight **W**_**i**_(**i** = **1**, **2**, **3**) is a 192 × 1 vector and each ${\text{w}}_{\text{i}}^{\text{j}}$ corresponds to one feature map **fm**^**j**^, which has 32 weight numbers corresponding to the channels. Thus, a high or low value of weight indicates the importance of the channels for specific forearm movements. All six feature maps contribute to the computation of joint angles with the FC layer weight. To check the motion pattern using weight, we can separate the FC layer weight into six 32 × 1 weight parts corresponding to feature maps and perform superposition calculation as a 32 × 1 vector. We then arrange the 32 weight numbers based on the 32 channels positions as the expected geometry plot. To analyze the WF/WE motion, we plotted a geometry plot based on **W**_**1**_; for P/S, we used **W**_**2**_; and for HG/HO, we used **W**_**3**_ (see Results Section and Figure 6).

## Results

### Five-Fold Cross Validation Results

The model used for training was created using pytorch1.3.1, GeForce RTX 2080 GPU, and CUDA10.1. For the training, we used the Adam optimizer to update the model parameters to minimize the loss function and mean-square error (MSELoss) as our loss function. The learning rate was 0.001. In the initial experiment, the training epoch was 15; For second experiment dataset, training epoch was 5 for transfer learning.

**Figure 7:**
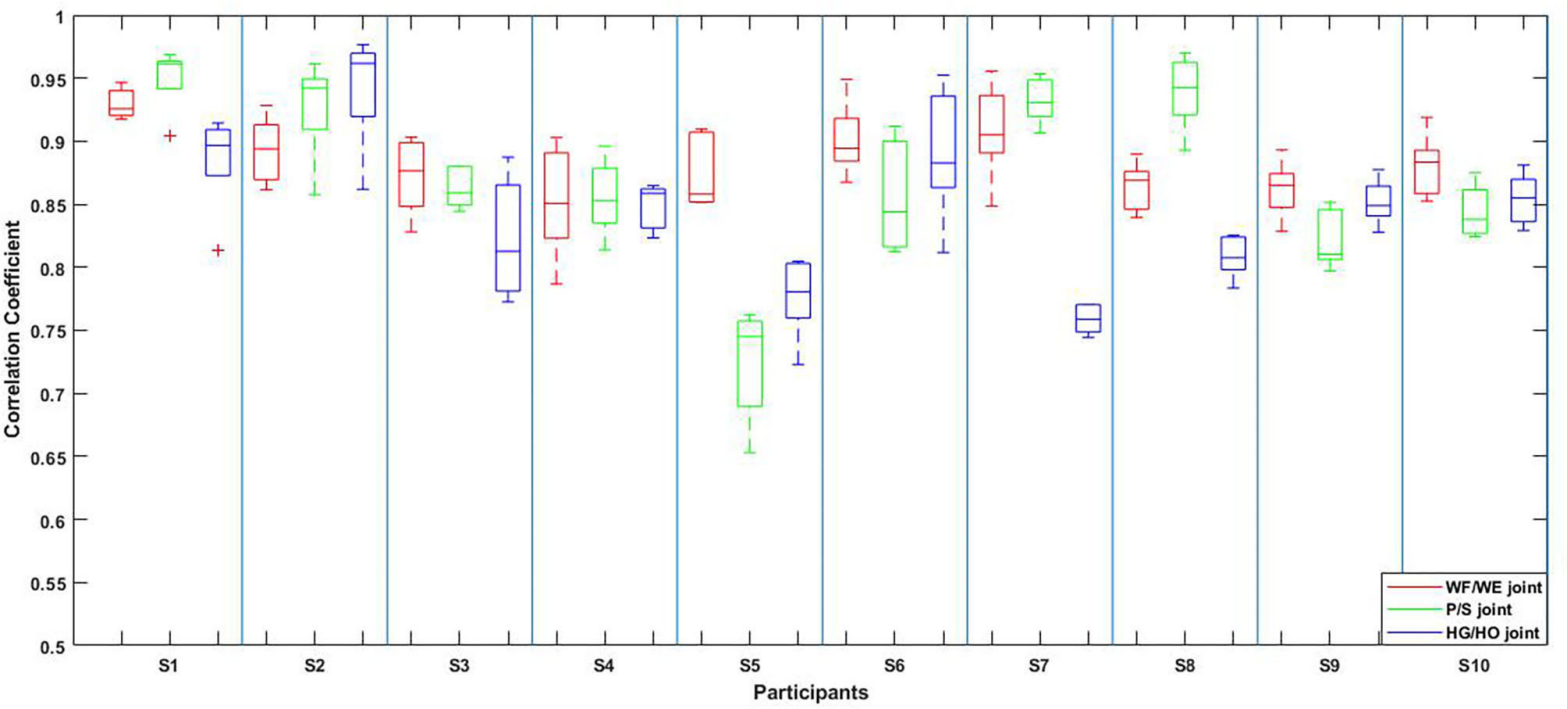
Box plot of five-fold CV results of all the participants according to Table 2. The red box is the CC result of WF/WE joint angles, the green box is the CC result of P/S joint angles, and the blue box is the CC result of HG/HO joint angles.

**Figure 8:**
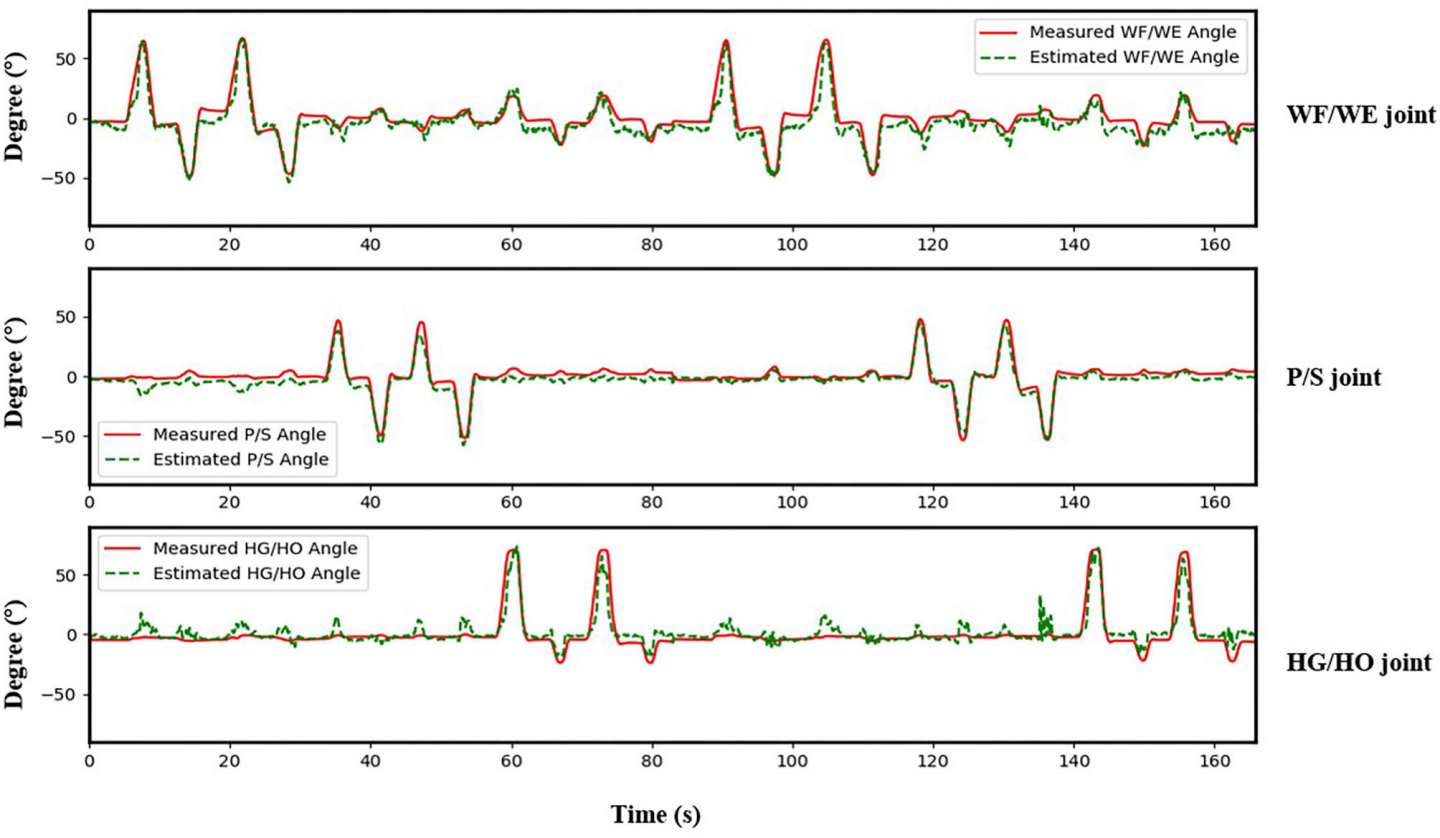
Testing result of participant S1. Top: WF/WE joint; Middle: P/S joint; Bottom: HG/HO joint. Red solid line is measured joint angles using Perception Neuron Motion Capture system. Green dashed line is estimated joint angles using proposed regression model. This result shows CC = 0.9465 for WF/WE joint angles, CC = 0.9686 for P/S joint angles, and CC = 0.9074 for HG/HO joint angles.

The result of the five-fold CV was evaluated using the CC. The mean CC values of all participants are listed in the left part (initial experiment) of Table 2, and Figure 7 shows the box plot corresponding to Table 1. An example of the comparison between the estimated joint angles using the proposed regression model and measured joint angles via the Perception Neuron Motion Capture system are plotted in Figure 8, where the red solid line represents the measured angles and green dashed line indicates the estimated angles. This figure shows the prediction result of S1, CC = 0.9465 for WF/WE joint angles, CC = 0.9686 for P/S joint angles, and CC = 0.9074 for HG/HO joint angles.

**Table 2:** CC results of five-fold cross validation (mean CC ± std).

	**Initial Experiment**			**Second Experiment**		
	**WF/WE**	**P/S**	**HG/HO**	**WF/WE**	**P/S**	**HG/HO**
S1	0.9298 ± 0.0121	0.9500 ± 0.0259	0.8849 ± 0.0410	0.9746 ± 0.0089	0.9189 ± 0.0273	0.9183 ± 0.0292
S2	0.8928 ± 0.0269	0.9266 ± 0.0405	0.9413 ± 0.0465	-	-	-
S3	0.8721 ± 0.0311	0.8628 ± 0.0164	0.8229 ± 0.0488	-	-	-
S4	0.8525 ± 0.0457	0.8556 ± 0.0311	0.8484 ± 0.0186	-	-	-
S5	0.8755 ± 0.0298	0.7235 ± 0.0459	0.7765 ± 0.0331	-	-	-
S6	0.9017 ± 0.0271	0.8563 ± 0.0406	0.8915 ± 0.0485	0.9210 ± 0.0243	0.8434 ± 0.0712	0.9298 ± 0.0443
S7	0.9087 ± 0.0355	0.9323 ± 0.0169	0.7589 ± 0.0105	0.9247 ± 0.0106	0.9647 ± 0.0080	0.8864 ± 0.0310
S8	0.8636 ± 0.0178	0.9392 ± 0.0269	0.8086 ± 0.0152	0.9050 ± 0.0372	0.9507 ± 0.0259	0.8577 ± 0.0404
S9	0.8617 ± 0.0210	0.8223 ± 0.0213	0.8519 ± 0.0164	-	-	-
S10	0.8799 ± 0.0231	0.8445 ± 0.0191	0.8540 ± 0.0186	0.9107 ± 0.0272	0.9464 ± 0.0281	0.8665 ± 0.0398
Mean	0.8838 ± 0.0270	0.8713 ± 0.0284	0.8439 ± 0.0297	0.9272 ± 0.0216	0.9248 ± 0.0321	0.8918 ± 0.0369

*“-” means this participant did not participate in the second experiment.

**Figure 9:**
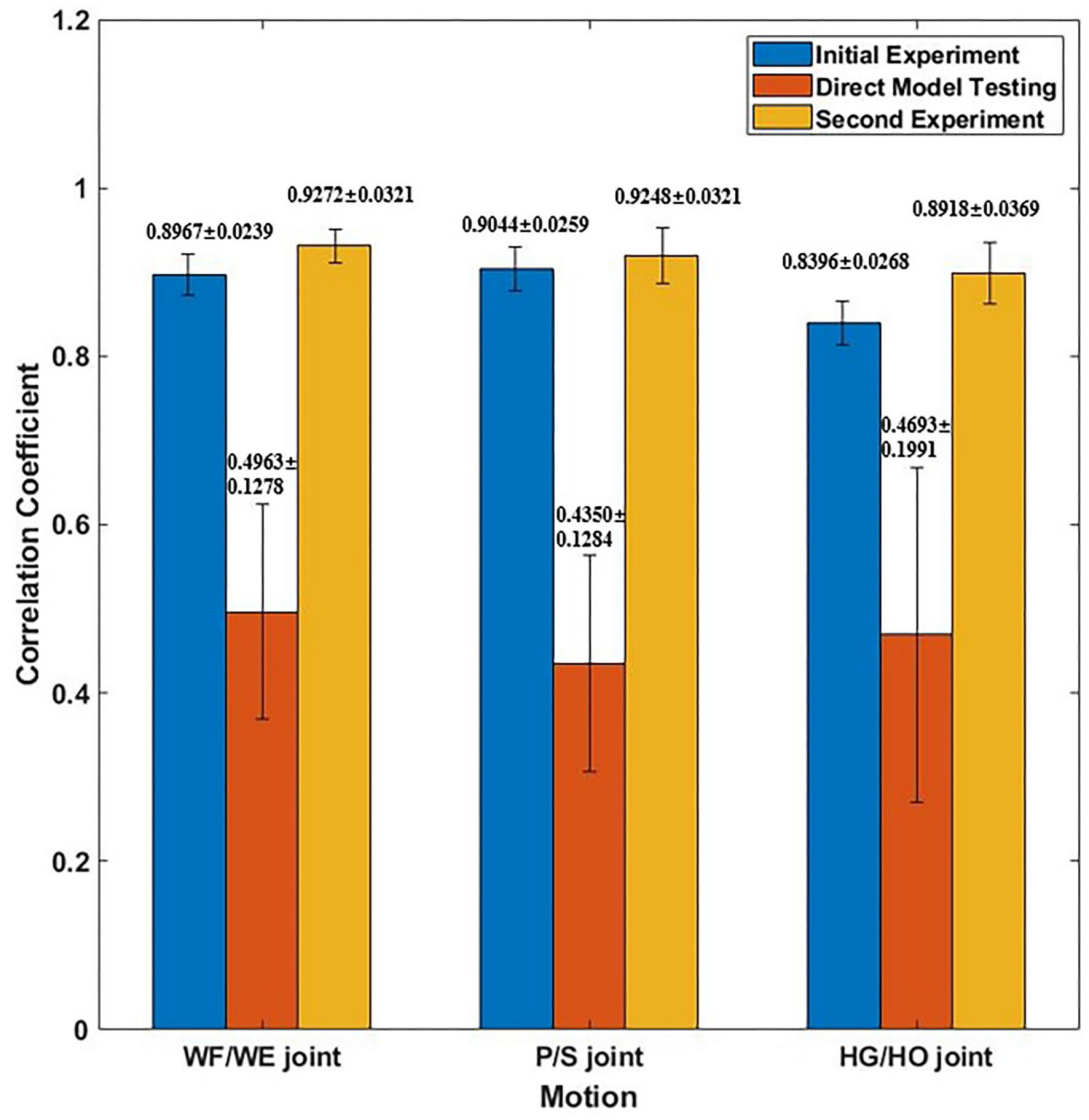
Mean CC result of participant S1, S6, S7, S8 and S10. Blue bar shows the mean CC result in initial experiment (15 epochs); Red bar shows the mean CC result of direct model testing, that is to use the trained model in initial experiment to test on the new dataset directly; Yellow bar shows the mean CC result in second experiment, which fixed the first layer and only to train FC layer (five epochs).

**Figure 10:**
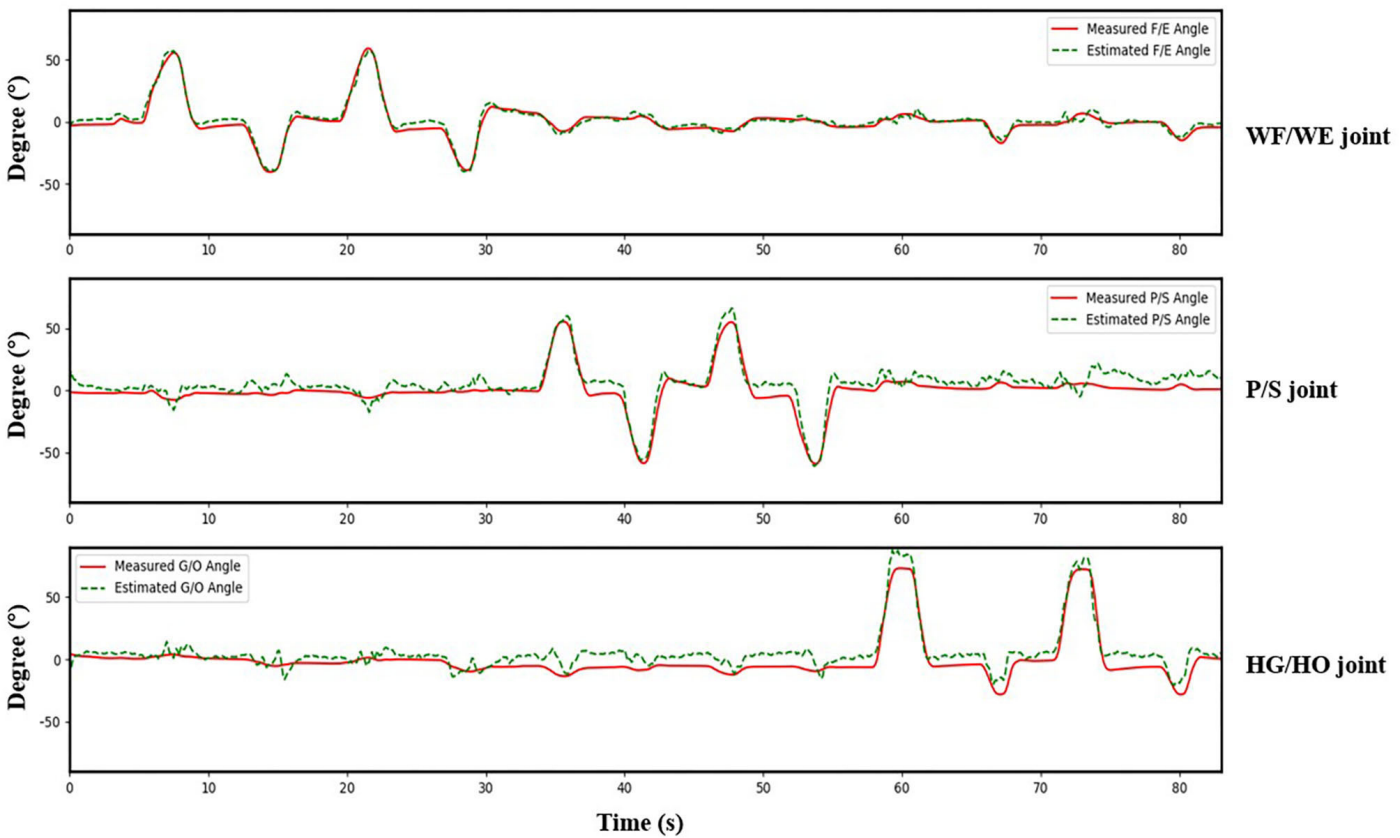
A testing result of participant S1. Red solid line is measured joint angles using Perception Neuron Motion Capture system. Green dashed line is estimated joint angles using proposed regression model. This result shows CC = 0.9793 for WF/WE joint angles, CC = 0.9553 for P/S joint angles, and CC = 0.9573 for HG/HO joint angles.

We invited the participant S1, S6, S7, S8 and S10 to complete the second experiment. The second experiment was conducted two months after the initial experiment. We used the trained model to test the new dataset directly to check the mean test CC, we called this procedure as *Direct Model Testing*. As we expected, the mean CC values are lower than the CC value in initial experiment respectively (Figure 9, red bar), this is because the magnitude and quality of the sEMG signal often differ grately in another day. Then, we used the trained model and fixed the convolutional layer, using transfer learning to check the model via five-fold CV. The average CC results are listed in the right part (second experiment) of Table 2. A comparison of the mean CC results in initial experiment, direct model testing and second experiment is shown in Figure 9, blue bar shows the mean CC value in initial experiment, red bar shows the direct model testing result, and the yellow bar shows the mean CC value in second experiment. An example of the comparison between the estimated joint angles using the proposed regression model and measured joint angles via Perception Neuron Motion Capture are plotted in Figure 10, where the red solid and green dashed lines represent the measured and estimated angles, respectively. The Figure 10 shows the prediction result of S1, CC = 0.9793 for WF/WE joint angles, CC = 0.9553 for P/S joint angles, and CC = 0.9573 for HG/HO joint angles.

### Model Performance Comparison

**Figure 11:**
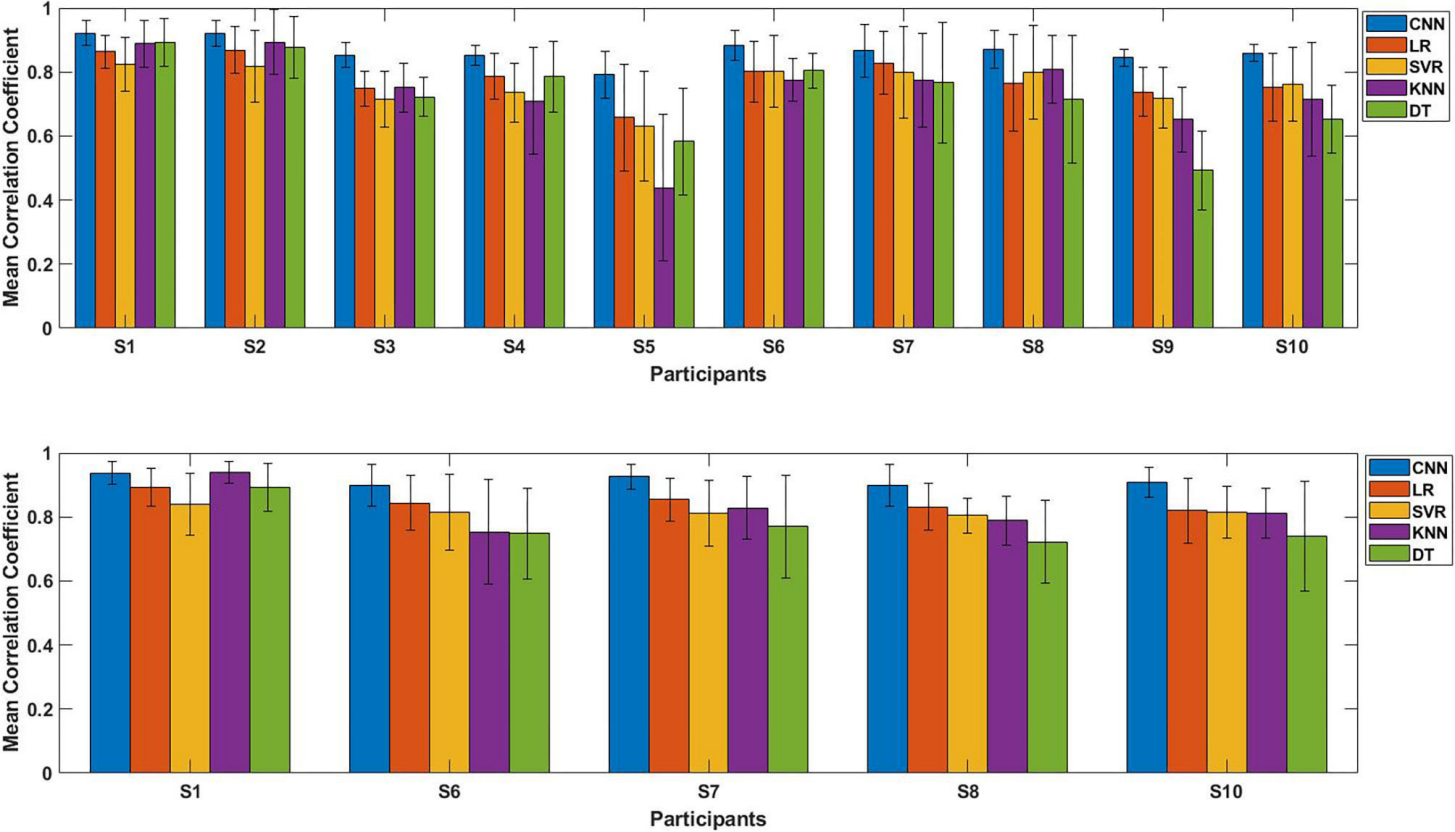
The performance comparison of each participants using five regression models (Proposed CNN, LR, SVR, KNN, DT). Top: Comparison result of initial experiment (without transfer learning; 10 participants); Bottom: Comparison result of second experiment (with transfer learning; five participants).

**Figure 12:**
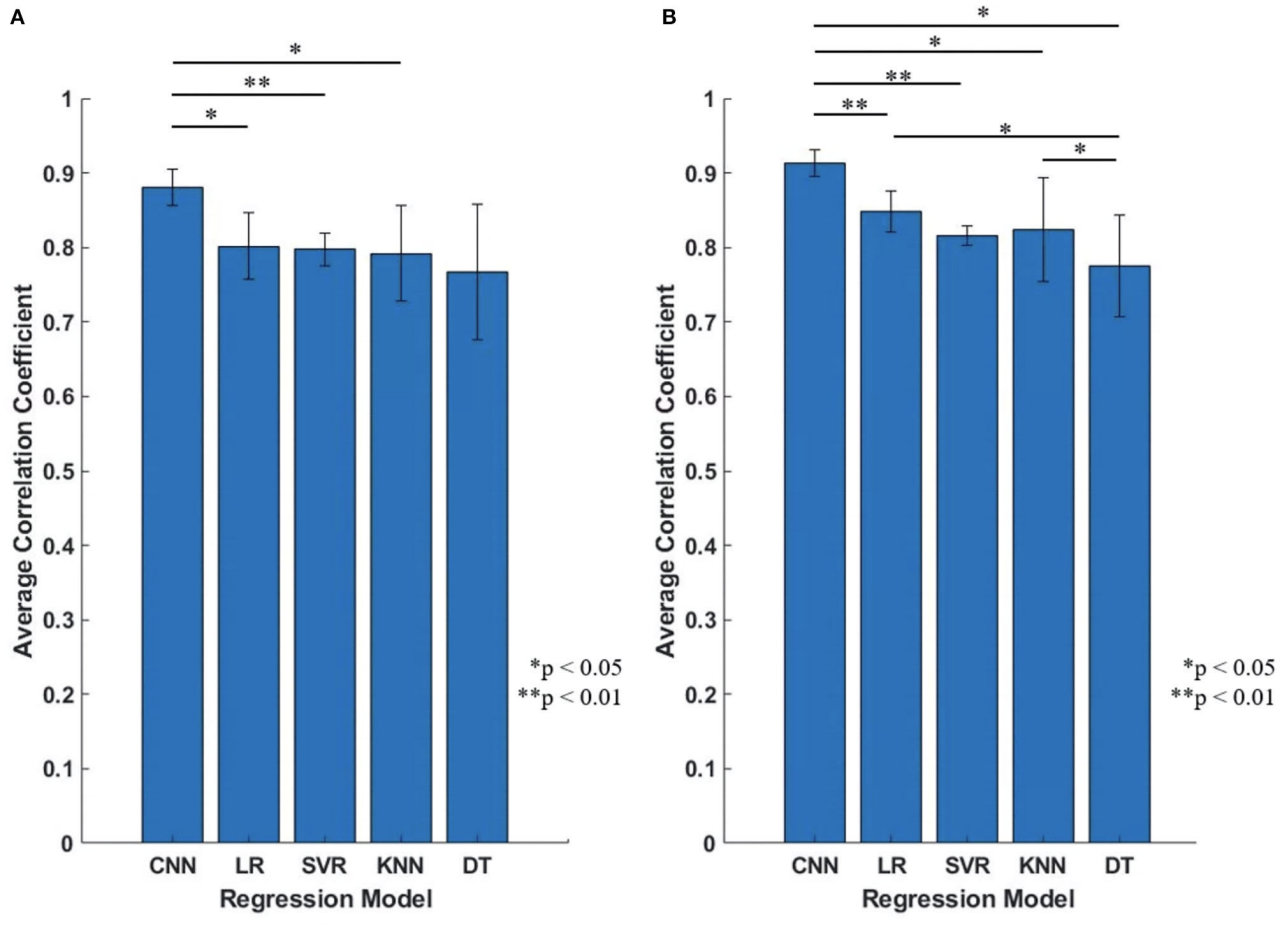
The performance comparison of the five regression models (Proposed CNN, LR, SVR, KNN, DT). **(A)** Initial experiment (without transfer learning; 10 participants); **(B)** Second experiment (with transfer learning; five participants). Statistical differences were calculated using Student's *t* test with Benjamini and Hochberg false discovery rate (BHFDR) correction for multiple comparison.

In order to highlight the advantages of proposed CNN-based regression model in joint angles prediction based on EMG signal, this model was compared with four conventional regression model: linear regression (LR), support vector regression (SVR), k-nearest neighbors (KNN) and decision tree regression (DT). We compared these five regression models with and without transfer learning, and the results were shown as Figures 11, 12. We will discuss the model performance comparison in the Section Model comparison.

Figure 11 shows the average CC results of the five regression models applied to each participant, the top figure is the comparison result of initial experiment (without using transfer learning; 10 participants), the bottom figure is the result of second experiment (with transfer learning; five participants). The results show that for any participant, whether transfer learning was applied or not, the proposed model outperformed the four conventional regression models.

Figure 12 is the comparison result between the five regression models, the vertical axis is the average CC of all participants, Figure 12A is the result of initial experiment (without using transfer learning; 10 participants) and Figure 12B is the result of second experiment (with transfer learning; five participants), average CC and standard deviation are shown as mean ± std format. We can find that for all participants, proposed CNN regression model outperformed than the other four conventional regression models, and the standard deviation of proposed CNN model is smaller and stable.

### Geometry Plot Results

Table 2; Figure 7 show that the proposed regression model accurately predicts the joint angles in the three DOFs of forearm motion. Therefore, we investigated the accurate model performance learnt directly from the filtered EMG signal and if the muscle activity can be analyzed from geometry plot. Different muscle areas perform different motions; therefore, we backtrack from the FC weight to create a geometry plot. In this section, we use the trained model trained obtained from all participants to find the correlation motion pattern. The corresponding result of participant S1 are shown in Figure 6 (result of all 10 participants can be found in Supplementary Material), each geometry plot includes two parts: the left side denotes the wrist flexor side and right side denotes the wrist extensor side. The color bar ranges from −1 to 1, the blue part shows −1–0, i.e., the area contributes to negative joint angles (WE, S, HO); the red part shows 0–1, i.e., the area contributes to positive joint angles (WF, P, HG); and the white part is 0, i.e., the area has no contribution to the corresponding motion. The green circle shows positive joint angles such as WF, P, and HG, and the purple circle shows negative joint angles such as WE, S, and HO.

## Discussion

We proposed a CNN-based regression model for real-time prediction of joint angle of wrist and hand motion using sEMG signals. We used the CC value to evaluate the model training effect for 10 participants in initial experiment and explore the learnability of this model by directly using EMG signals. Further, we backtracked the FC layer weight to create a geometry plot for the wrist flexor and wrist extensor sides, and checked the area of the muscles for the corresponding motions. We hope that the model can be robust and applied in different day, participant S1, S6, S7, S8 and S10 were invited to repeat our experiment for smaller number of datasets as second experiment. Firstly, we tested the new dataset directly using the existing trained model to prove the prediction accuracy reduced in another day. Then, we applied transfer learning, fixed the first layer, updated the FC layer parameters to train the new smaller dataset to check the testing CC result and the geometry plot, so that to verify an improvement in the testing CC result. We also compared proposed CNN model with four conventional regression model (LR, SVR, KNN and DT) to prove the superiority of the proposed model. The results showed the model can be used in different day with small number of sEMG data using transfer learning.

### Convolutional Layer Design

In this study, we proposed a model with two layers: a convolutional layer and an FC layer. The function of the FC layer is to flatten the feature maps into a single vector; hence, the key point in designing the CNN-based regression model is the convolutional layer. Sakhavi et al. (2018) considered three types of convolution kernel for linear mixture of EEG signal input, including CW-CNN, channel mixing CNN (CM-CNN), and channel-wise convolution with channel mixing (C2CM). According to Sakhavi et al. (2018) research, the difference between CW-CNN, and CM-CNN and C2CM is that CW-CNN does not demonstrate channel mixing. Channel mixing leads to a widened network, and without channel mixing, the receptive field of the network is emphasized.

The process of skeletal muscle contraction and relaxation can be expressed as follows: the electrical signals from the brain reach the nerve endings, and the action potentials are transmitted to the cell membranes of the nerve endings. A series of chemical changes occur to change the conformation of tropomyosin and expose muscle movement. The binding site of the protein and myosin, and head of myosin is activated generating power to swing the head and slide the thin filaments. Since the arrival of electrical signals at the neurons to contract or relax a muscle, a time delay should be generated at times. Thus, the input data in our study is a linear mixture of 32 EMG channels with temporal dimension, and we hope that the time period input can provide sufficient information of the sEMG signal and muscle force pattern that occur during the time delay.

The channel-wise filter (CW-CNN) designed in the convolutional layer can reduce the temporal dimension to one-dimensional feature maps, including 32 channels information. Thus, the channels corresponding to each number from the feature maps are independent of each other. Further, because the temporal dimension is reduced, we obtained the muscle force pattern from each channel using such a channel-wise convolution kernel. Unlike the CW-CNN proposed in Sakhavi et al. (2018), which was used to process the EEG signal, we used a one-dimensional convolution kernel to obtain the muscle force pattern, which is called as a *force pattern filter* (FP filter).

### Five-Fold Cross Validation

In the initial experiment, we used the five-fold CV to train and test the proposed model. For each participant, we created five groups of datasets, we trained the four groups and tested them on the remaining dataset. Hence, there were five CC results for each participant. Equation (3) was used to calculate the average values of the CC of different participants for joint WF/WE, joint P/S, and joint HG/HO. Table 2 left part (initial experiment) shows the five-fold CV results of all participants in the form of mean ± std. This result is plotted in Figure 7. After checking the raw sEMG signal from each participant, the qualities of the sEMG signal of S1, S2 and S8 are the best, S3, S4, S6, S7, S9 and S10 are slightly noisy, and sEMG signal quality of S5 is the worst and noisy channels are more than that of the other four participants. Therefore, we inferred that several noisy channels confuse our model in predicting P/S joint angles and HG/HO joint angles, which contain more inner muscle sEMG signals, and hence are more difficult to predict than the WF/WE joint angles. We can further conclude that with a less noisy sEMG signal as input, the proposed regression model can perform very well in predicting the three important DOF joint angles. Figure 8 shows one of the testing results obtained for the five-fold CV of S1. The top figure shows the WF/WE joint, middle figure shows the P/S joint, and bottom figure shows the HG/HO joint. We found that the predicted joint angles met our expectations. When the participant performed hand grip and open motion, the WF/WE joint showed small angles and vice versa. Further, the HG/HO joint showed a smaller angle when performing wrist flexion and extension motion, which is appropriate because WF/WE and HG/HO have common muscle areas. The participants S1, S6, S7, S8 and S10 with relatively high-quality sEMG signals (Figure 3 shows the raw sEMG signal of S1 from one of the channels) participated in the second experiment.

While the second experiment, with the same experimental paradigm (Figure 4), we obtained five trials dataset, and each trial data was regarded as one group of datasets to continue with the five-fold CV training and testing. If we used a trained model for prosthetic hand control, the prediction accuracy decreases in another day. Furthermore, even for the same participant, the quality of the sEMG signal always changes, with the existing trained model, the testing result in another day should be worse; hence, amputees should train the model to calibrate the control system before using the prosthetic hand. We used the existing model to do transfer learning, the first layer (convolutional layer) was fixed, and the testing CC result of the five-fold CV of the five participants is shown in Table 2, where the mean CC of the five participants of the WF/WE, P/S, and HG/HO joints was 0.9272 ± 0.0216, 0.9248 ± 0.0321, and 0.8918 ± 0.0369, respectively.

Figure 9 shows the corresponding result, the blue bar is the mean testing CC result of S1, S6, S7, S8 and S10 in initial experiment from 10 trial dataset and trained using five-fold CV; the red bar is the testing CC result in Direct Model Testing, which means to test the existing trained model on new dataset directly; the yellow bar is the testing result of S1, S6, S7, S8 and S10 in second experiment, fixed the convolutional layer and trained FC layer in five epochs, as shown in the right part of Table 2. When compared to the blue bar, which indicates the initial experimental result, the red bars show that the mean CC value of the participants in Direct Model Testing lower than in Initial Experiment, WF/WE joint CC reduced to 0.4963 ± 0.1278 from 0.8967 ± 0.0239, P/S joint CC reduced to 0.4350 ± 0.1284 from 0.9044 ± 0.0259, HG/HO joint CC reduced to 0.4693 ± 0.1991 from 0.8396 ± 0.0268. This result is in line with our expectations. With small number dataset and only five epochs, the mean CC results are improved, the yellow bars show that, compared to the red bars which indicates Direct Model Testing, WF/WE joint CC reaches to 0.9272 ± 0.0321, P/S joint CC reaches to 0.9248 ± 0.0321, HG/HO joint CC reaches to 0.8918 ± 0.0369. We can find that with smaller dataset and only 5 trials, the model can keep the high CC value every day, and even higher than before. Let us compare yellow bar (second experiment) to blue bar (initial experiment), average CC of WF/WE joint, P/S joint and HG/HO joint were improved. The Figure 10 shows a testing result of the five-fold CV of S1 in second experiment, top figure shows WF/WE joint, middle figure shows P/S joint, and bottom figure shows HG/HO joint.

Then we will discuss the reason why using transfer learning can improve the performance (Figure 9). In the initial experiment, parameters from both FP filter and FC layer were trained from the 10 trails datasets of each participant, the parameters of the model include the information of all of the dataset. As we discussed in Section convolutional layer design, reference to CW-CNN (Sakhavi et al., 2018), the proposed FP filter designed in the first convolutional layer can be used to obtain the muscle force pattern from each channel, namely, the feature map extracted from the EMG signal input should be force pattern, and different people have their own force pattern. In the second experiment, if we fix the convolutional layer (FP filter) and only update FC layer parameters, the model still contains the previous 10 trial dataset information, and the FC layer parameters can adapt the model to the new data set. From the perspective of the entire training process, it is equivalent to adding a new dataset to the original dataset, totally 15 trial datasets. Therefore, this approach can ensure that in the daily update training, although there is only less training dataset, the model parameters always contain the training information of all previous datasets. The overall training dataset is constantly superimposed. The prediction result will become better, and it will also be more conducive to amputees to update the training daily.

### Model Comparison

To confirm the proposed CNN model performs well, we compared it with four conventional regression models (LR, SVR, KNN and DT). Figure 11 shows the results of each participants using different regression model with error bars, top figure is the initial experiment result (without transfer learning), bottom figure is the second experiment result (with transfer learning); Figure 12 shows the results of the model comparison on all participants with transfer learning (Figure 12A) and without transfer learning (Figure 12B). Both Figures 11, 12 show that the proposed model outperforms the four conventional models in the dataset of 10 participants. The comparison results also prove that the proposed model performs better in another day using transfer learning with even small amount of dataset and fewer training epoch. When measuring muscle activity, the magnitude and quality of the signal often differ greatly depending on the contact resistance between the electrode and the skin. Therefore, in the trained model, there is a problem that the estimation accuracy of the data on another day is lowered. In order to solve this problem, we aimed to improve the estimation accuracy even with a small amount of data on new day. The average CC still shows the highest compared to the four traditional regression model with transfer learning (Figure 12B).

As presented in Figure 12, the statistical analysis was performed to show the proposed CNN shows significantly higher performance than the conventional methods. Statistical differences were calculated using Student's *t* test with Benjamini and Hochberg false discovery rate (BHFDR) (Benjamini and Hochberg, 1995) correction for multiple comparisons. From Figure 12A, the CC value for the proposed CNN is 0.8803 ± 0.0247, is significantly higher than LR (*p* = 0.021 <0.05), SVR (*p* = 0.0016 <0.01) and KNN (*p* = 0.0337 <0.05); From Figure 12B, the CC value for CNN is 0.9133 ± 0.0175, is significantly higher than LR (*p* = 0.0044 <0.01), SVR (*p* = 0.0004 <0.01), KNN (*p* = 0.031 <0.05), and DT (*p* = 0.015 <0.05). In our CNN model, we only used one convolutional layer to make the model have better performance than conventional regression models, instead of using multiple convolutional layers or more complex deep learning models to achieve high-precision predictions, which makes regression prediction for joint angles with higher efficiency.

### Motion Pattern

**Figure 13:**
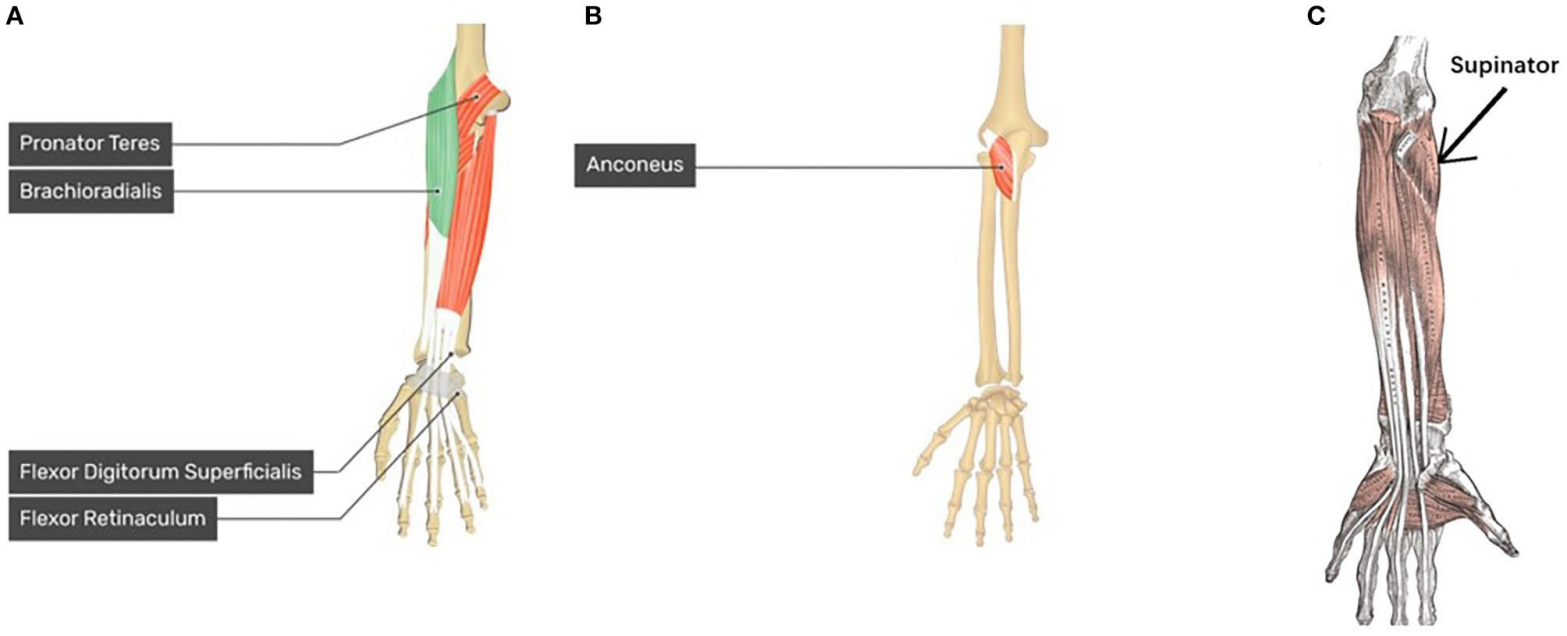
Forearm and Hand anatomy for motion pattern analysis. **(A)** Anatomy of brachioradialis which related to WF motion, pronator teres which related to P motion, and flexor digitorum superficialis (FDS) which related to HG motion (left hand) (GetBodySmart2021, 2021); **(B)** Anatomy of anconeus which related to WE motion (left hand) (GetBodySmart2021, 2021); **(C)** anatomy of supinator related to S motion (left hand) (Kenhub, 2021).

In this study, we assumed that the proposed regression model can obtain the corresponding motion pattern through the sEMG signal of different channels on the wrist muscle. After the FP filter in convolutional layer, the feature maps include the force pattern information of the participant, then the FC layer interacts directly with the feature maps to calculate the joint angle output, we thought that it should be motion pattern. Actually, the motion pattern showed as geometry plot in different muscle area conforms to anatomy. Figure 6 shows the geometry plot of participant S1, and the participants' geometry plots can be checked from Supplementary Material. Each geometry plot includes the flexor side (left) and extensor side (right). In the actual multi-array electrode sleeve, the left border of the flexor side is connected to the right border of the extensor side, and the right border of the flexor side is connected to the left border of the extensor side. The WF/WE geometry plot was constructed using **W**_**1**_ from FC layer and those of the P/S and HG/HO using **W**_**2**_, **W**_**3**_, respectively. The circles show the motion pattern position; the green circle shows the motion pattern corresponding to the positive joint angles and purple circle shows the motion pattern corresponding to negative joint angles. In the geometry plot, the matrix shows the motion pattern color with the channel index. The geometry plots of the participants (Figure 6; Supplementary Material) show that all participants show similar motion patterns, although the channels are not the same, and the adjacent area can display the muscle activity. This is because the different sizes of the participant's forearm and dislocation of the multi-array electrode sleeve may lead to this result. Using the anatomy of the forearm (Figure 13), the motion patterns can be discussed as follows.

- WF/WE joint: When WF/WE motion is performed, both sides show WF or WE motion patterns. This is an example of muscle contraction and muscle relaxation during muscle activity. S1 and S2 show almost the same motion pattern, and Table 2 shows that the two participants demonstrate best CC results with the WF motion occurring on channels 13, 21, 29, and 28 near channel 24, while WE motion occurs on channels 6, 14, 12, 20, 25, and 26. S3–S10 show similar patterns but near the above area. For S5, channel 17 is the adjacent area of channel 24; hence, it is a similar motion pattern when compared with the WF pattern shown in S1 and S2 (in this pattern, S3 and S4 shown in channels 15 and 16 represent dislocation of the sleeve).
- P/S joint: When P/S motion is performed, both sides show wrist pronation or supination motion pattern, which is an example of muscle contraction and muscle relaxation during muscle activity. The P/S motion generated from deep layer muscle is compared to WF/WE. The motion pattern may not be representing the correct anatomy of the P/S motion activity in the forearm; however, they show a similar motion pattern.
- HG/HO joint: The difference between the previous motions (WE/WF and P/S) is that HG/HO motion is generated from forearm motion and deeper muscles that lead to finger motion (we can regard hand grip and open as finger motion). The geometry plot shows that the FC layer weights of each participant are quite different. However, we can find a similar motion pattern area from Supplementary Material.

Moreover, according to human anatomy (Figure 13) and comparing with Figure 6, we can discuss the motion pattern using the anatomy of the forearm muscles:

- The WF motion produced by the brachioradialis of the forearm (Figure 13A) and WE motion produced by the anconeus of the forearm (Figure 13B) correspond to the area of channels 13, 21, 29, and 28 (brachioradialis) and the area of channels 25 and 26 (anconeus), respectively. By comparing Figure 6 with Figures 13A,B, we found that the motion pattern is correct. We may consider another pattern area, such as channels 23, 24, 15, 16, and 6, owing to muscle contraction and relaxation. When we perform the WF and WE motion, these aforementioned areas show have motion activity.
- P motion produced by pronator teres of the forearm (Figure 13A) and S motion produced by the supinator of the forearm (Figure 13C) correspond to the area near channel 22 and 30 (pronator teres), and the area of channels 13, 21, and 29 (supinator), respectively. Comparing Figure 6 with Figures 13A,C show that the motion patterns are similar to the anatomy results. Because P/S motion contains the interactive movement between two bones, the motion generates the muscle activity on the opposite side, such as channels 17, 18, or 25 (P) and channels 16 or 11 (S). Thus, we consider the motion patterns to be appropriate.
- HG motion is produced mainly by the flexor digitorum superficialis (FDS, Figure 13A) and muscles that produce WF, such as brachioradialis. Similar to HG, the HO motion is a complicated motion produced by many muscles, including the anconeus area of the WE. The FDS corresponds to the area around channels 14 and 15 or the adjacent channels. Figure 6 shows that the motion patterns of the HG of participant S1 are near the FDS area. The patterns occur in the area near channels 24 and 16 (near the WF pattern area). It is difficult to evaluate the motion pattern of HO, but the HO motion pattern shows the area mainly on the extensor side. When the HO motion was performed, these areas show motion activity; thus, we infer that the motion pattern is appropriate.

### Limitation of Our Work

In this work, we used a window of 500 ms to segment sEMG signal data flow as CNN input for offline analysis, and we got high CC result after training the regression model using such window length. However, we did not apply it for real-time control. In real-time control, the estimation window lengths should range from 50 ms to 400 ms (Hargrove et al., 2009), 500 ms window might generate delay, we will reduce the window size in our next topic.

## Conclusion and Future Work

In this study: (1) We proposed a CNN-based regression model to estimate 3-DOFs joint angles (WF/WE and P/S as wrist motion, and HG/HO as finger motion) based on sEMG signal, and it performed the highest when we compared it to another regression models; (2) We used transfer learning with small amount of new dataset to make the model can be calibrated in another day. The model comparison result shows that, compared to LR, SVR, KNN and DT, proposed CNN model significantly performs higher than conventional models with and without transfer learning; (3) We tried to find the reason why the proposed model can learn the motion information from muscle, so we design the convolutional filter as CW-CNN filter to obtain force pattern as feature maps, and we tracked back to check the geometry plots to analyze the motion patterns.

In our future work, we would use this model to predict the mentioned 3-DOFs joint angles (WF/WE, P/S and HG/HO) in real time and send the predicted joint angles to the prosthetic hand control system to achieve real-time control of the prosthetic hand.

## Data Availability Statement

The raw data supporting the conclusions of this article will be made available by the authors, without undue reservation.

## Ethics Statement

The studies involving human participants were reviewed and approved by the ethics committee of the Tokyo Institute of Technology. The patients/participants provided their written informed consent to participate in this study.

## Author Contributions

ZQ designed research and experiment, collected data, analyzed the results and completed the paper manuscript. ZH helped how to use the experimental equipment Perception Neuron for data acquisition, and data collection. SS, NY, and YK made discussion together, analyzed the results, and reviewed manuscript. All authors contributed to the article and approved the submitted version.

## Conflict of Interest

The authors declare that the research was conducted in the absence of any commercial or financial relationships that could be construed as a potential conflict of interest.

## Publisher's Note

All claims expressed in this article are solely those of the authors and do not necessarily represent those of their affiliated organizations, or those of the publisher, the editors and the reviewers. Any product that may be evaluated in this article, or claim that may be made by its manufacturer, is not guaranteed or endorsed by the publisher.
