# Supplementary figures and images for: Multi–Joint Angles Estimation of Forearm Motion Using a Regression Model
Source: Front Neurorobot. 2021 Aug 2;15:685961. doi: 10.3389/fnbot.2021.685961 (PMC8366416; doi:10.3389/fnbot.2021.685961)

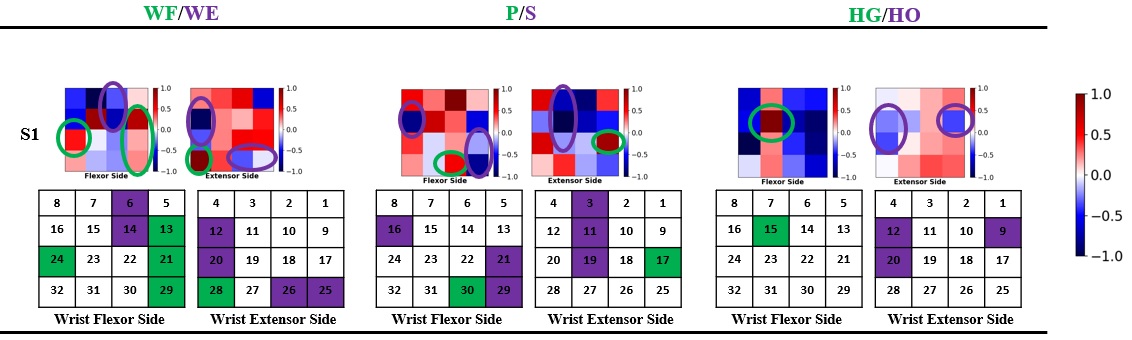

Supplement: Supplementary file 1 [file Data_Sheet_1.ZIP › Geometry Plot/S1.jpg]

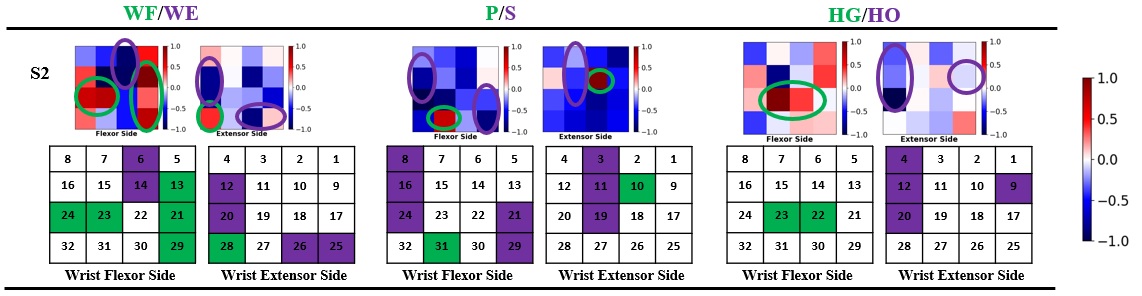

Supplement: Supplementary file 1 [file Data_Sheet_1.ZIP › Geometry Plot/S2.jpg]

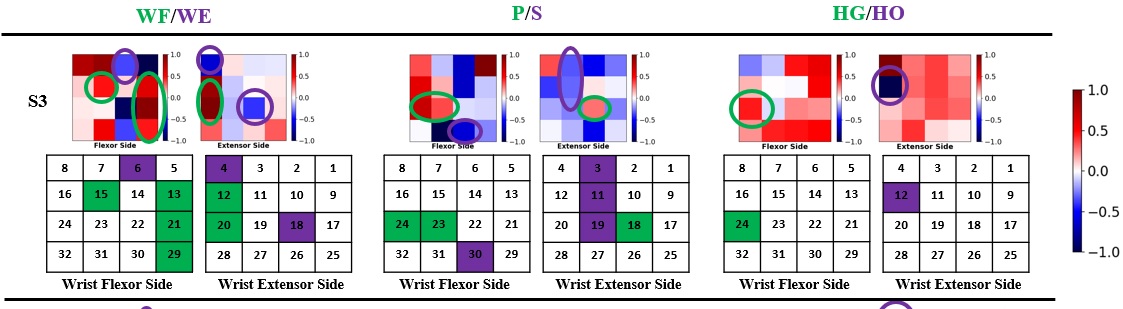

Supplement: Supplementary file 1 [file Data_Sheet_1.ZIP › Geometry Plot/S3.jpg]

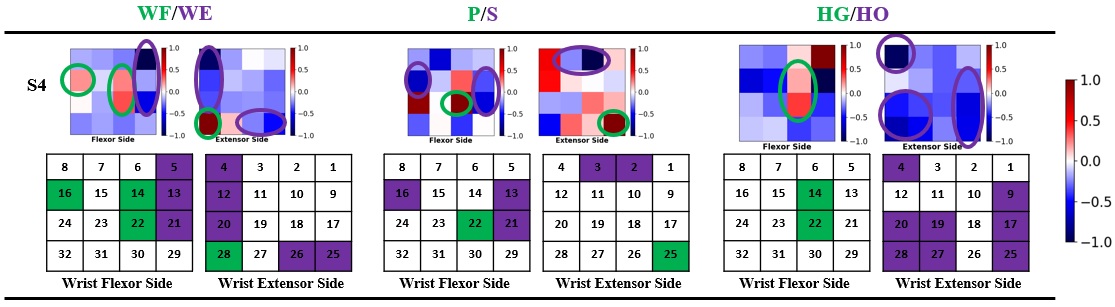

Supplement: Supplementary file 1 [file Data_Sheet_1.ZIP › Geometry Plot/S4.jpg]

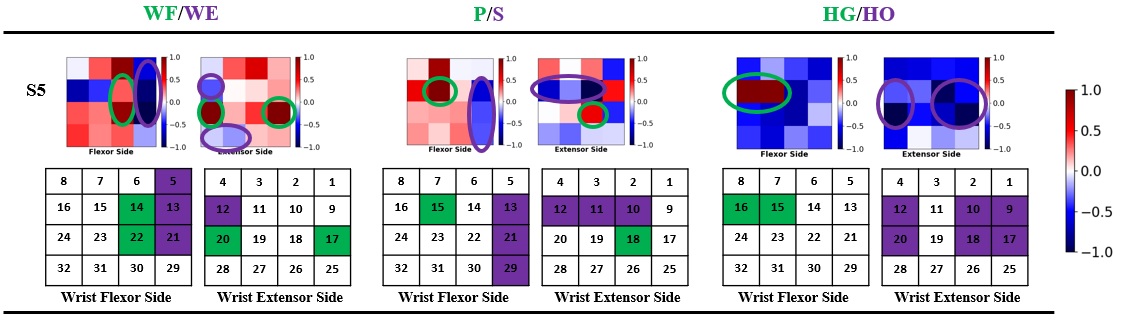

Supplement: Supplementary file 1 [file Data_Sheet_1.ZIP › Geometry Plot/S5.jpg]

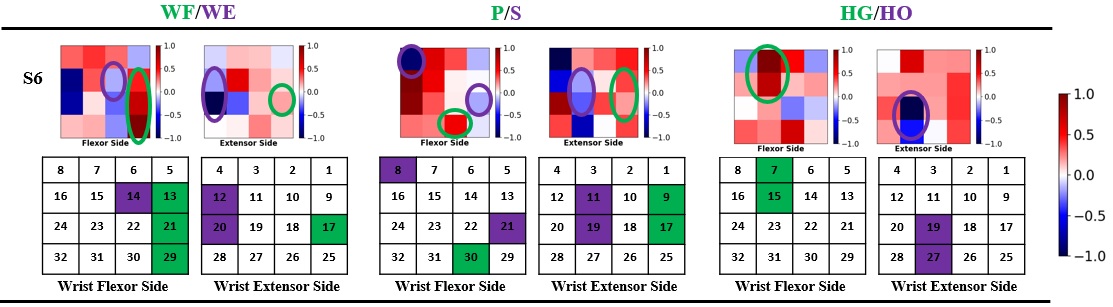

Supplement: Supplementary file 1 [file Data_Sheet_1.ZIP › Geometry Plot/S6.jpg]

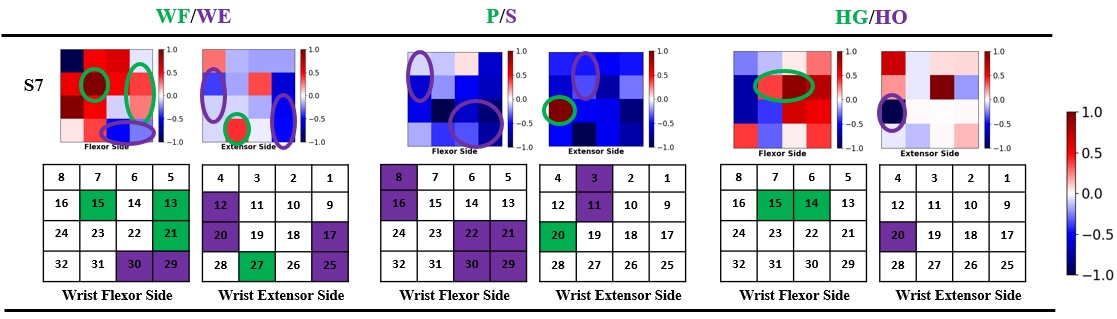

Supplement: Supplementary file 1 [file Data_Sheet_1.ZIP › Geometry Plot/S7.jpg]
